# Effect of community based nutritional education on knowledge, attitude and compliance to IFA supplementation among pregnant women in rural areas of southwest Ethiopia: a quasi experimental study

**DOI:** 10.1186/s12889-023-16798-y

**Published:** 2023-10-05

**Authors:** Diriba Kumara Abdisa, Debela Dereje Jaleta, Dereje Tsegaye, Mohammedamin Hajure Jarso, Gemechis Dereje Jaleta, Gamachis Firdisa Tolesa, Keno Melkamu Kitila

**Affiliations:** 1 Department of Public Health, College of Health Science, Mettu University, Mettu, Ethiopia; 2 Department of Nursing, College of Health Science, Mettu University, Mettu, Ethiopia; 3 Department Psychiatry, Madda Walabu University, Shashemene Campus, Ethiopia; 4 Department of Medical Laboratory, College of Health Science, Mettu University, Mettu, Ethiopia

**Keywords:** Nutritional education, Knowledge, Attitude, Compliance, IFA supplementation

## Abstract

**Background:**

Compliance with the iron folic acid supplementation is low and not at the required level to prevent anaemia during pregnancy in many countries, including Ethiopia, even though an iron-folic acid supplementation program is being implemented. The aims of this study were to determine the effect of community-based nutritional education on knowledge, attitude, and compliance to IFA supplementation in Ilu Aba Bor zone of southwest Ethiopia.

**Method:**

A pretest–posttest quasi-experimental study design consisting of intervention and control group was conducted among pregnant women. The total sample size of 472, therefore, 236 pregnant women for each interventional and control group from 16 kebeles were randomly selected in two districts. A multi-stage sampling technique was used to select the study participants. Then, individual study units were selected using a simple random sampling technique and followed until the end of the study period. Effect of community-based nutritional education on knowledge, attitude, and compliance to IFA supplementation among pregnant women in rural areas were measured.

**Results:**

A total of 472 pregnant women participated in the study during the baseline and 437 (92.6%) were in the study until the end. The majority (49.2%) of respondents were 21–25 years of age, with a mean age of 23.4 (SD = 3.7) years. Community-based nutrition has resulted in a statistically significant increase in levels of maternal knowledge of IFAS by 15.2% in the intervention group compared to 5.1% in the control group. Similarly, the intervention group had odds of developing a positive attitude toward IFA 5.6 (4.01, 7.85) times higher than the control group. Moreover, in this study, the odds of compliance towards IFA supplementation were 3.9 (2.67, 5.57) times higher among those who received nutrition education than those women who did not.

**Conclusion:**

This study revealed that community-based nutritional education can result in a significant change in knowledge, attitude, and compliance towards IFA supplementation and supports the literature suggesting the importance of the intervention to overcome the problem of poor compliance and its associated consequences.

## Introduction

Globally, iron deficiency is the most widespread nutritional deficiency and the most common cause of anemia during pregnancy [1]. This is due to the increased demand for nutrients during these micronutrients. Therefore, Iron and Folic Acid (IFA) Supplementation is a key intervention for the prevention and control of anemia during pregnancy [2]. Iron supplementation, fortification of foods with iron, nutritional education, and deworming, done alone or in combination, are the common strategies for anemia prevention and control.

Iron-folic acid (IFA) supplementation during pregnancy was reported to result in a 70% reduction in anemia at term, a 67% reduction in iron deficiency anemia, and a 19% reduction in low birth weight incidences [1, 3]. The risk of neonatal mortality also decreased in infants whose mothers reported taking antenatal iron-folic acid supplements during pregnancy compared to those who did not. The World Health Organization (WHO) recommends initiating daily Iron and Folic Acid supplementation during pregnancy, a standard dose of 30–60 mg Iron and 400 g folic acid, as early as possible as a part of ANC programs for a positive pregnancy outcome [4].

Nearly 38% (32 million) of women in pregnancies are anemic in the world. Almost half, 46.3% (9.2 Million) of these are in Africa [5]. In sub-Saharan Africa, anemia during pregnancy accounts for 57%. In Ethiopia, the prevalence of anemia during pregnancy is estimated to be 29% [6].

IDA is a major risk factor for preterm delivery [7], stillbirth [8], and maternal death during childbirth. Additionally, anemic mothers were reported to give birth to a high proportion of low-birth-weight infants as compared to women with normal iron status [9]. Anemia in pregnancy also contributes to intergenerational cycles of poor growth in populations and cognitive impairment [10].

Even though an iron-folic acid supplementation program is being implemented in many countries, including Ethiopia, compliance with the IFA supplement is low and not at the required level to prevent anemia during pregnancy [6]. In Ethiopia, according to EDHS 2016, < 5% took an iron folic acid supplement for the recommended period (90 days or more), < 6% took 60–89 pills, about 30% took 60 pills, and around 58% did not take any iron tablets during their most recent pregnancy [11]. There are several factors responsible for not conforming to the recommended iron and folic acid supplementation during pregnancy, such as socioeconomic status, ANC utilization, knowledge towards IFA supplementation, attitude towards IFA supplementation, knowledge about anemia, maternal age, and previous illness [12]. While there is free provision of IFA supplementation, there is a need to scale up interventions to address poor compliance [13]. Therefore, there is an urgent need to address the factors affecting compliance and develop innovative strategies to mitigate them to increase IFAS coverage and eventually, substantially reduce the burden of pregnancy-related anemia for improved maternal and child outcomes [14].

Therefore, we are interested in testing the hypothesis that IFA supplements provided and monitored by government-supported community health workers will result in increased knowledge, attitude, and compliance levels during pregnancy compared to pregnant women who are supplemented by routine antenatal care methods. A study conducted in Kenya found that nutritional education was a major factor in reduced compliance with IFA among pregnant women and suggested the need for educating mothers during ANC follow-up or other possible mechanisms. Although they may have a chance to hear about IFA, a significant proportion of them have inadequate information about its benefits. This may arise from the quality of health education delivered to them during antenatal follow-up [15]. Community-based nutritional education may improve compliance with IFA supplementation, subsequently reducing anemia among pregnant women. The claim is based on the fact that community agents are able to reach pregnant women through home visits to provide IFA supplements, counseling, referrals, and follow-up. Hence, this study aimed to assess the effect of community-based nutritional education on knowledge of IFA supplementation, attitude, and compliance level among pregnant women in rural communities in Ilu Aba Bor Zone, southwest Ethiopia.

## Methods

### Study setting, design, and participants

This study was conducted in the Ilu Aba Bor Zone, which is located in the southwest of Ethiopia. A zonal town, Mettu is 600 km away from the capital, Addis Ababa. The zone has one town administration and fourteen rural districts, with a projected total population of 1,606,502. One referral hospital and one district hospital are found in the zone, serving the population of the zone. A pretest–posttest quasi-experimental study design consisting of an intervention and control group was used. Baseline and end-line data were collected in both the control and intervention groups. The intervention group received weekly nutrition education and counseling, while the control group did not. The study population consisted of all randomly selected pregnant women in selected kebeles (the lowest administrative unit) in the Ilu Aba Bor zone. All pregnant women with gestational ages less than 20 weeks were included and pregnant women with evidence of mental impairment or who were seriously ill were excluded from the study.

**Fig. 1:**
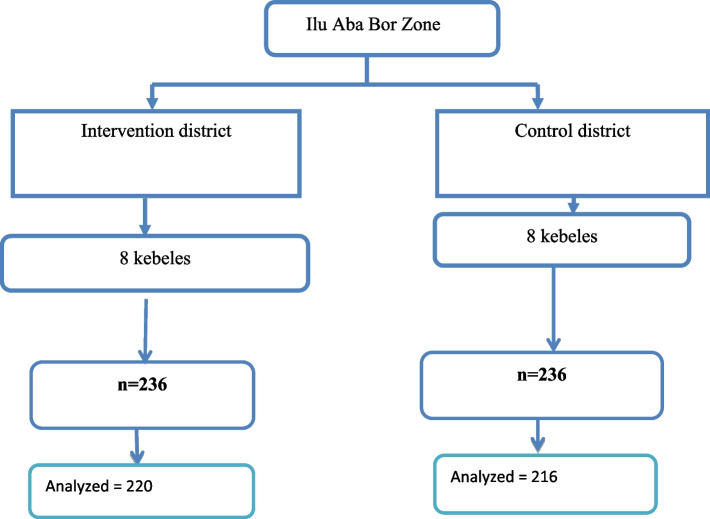
Flow diagram of study participants

To come up with the final sample size, separate samples were calculated for each of the objectives, and the largest sample was taken to enroll the study participants to increase power and level of precision. All the sample sizes were determined using G*Power 3.0.10. The following assumptions were generally considered when estimating the required minimum sample sizes for the objectives: a confidence level of 95%, a power of 80%, and a margin of error of 5%. The 95% confidence level implies 5% type I error (α), and the 80% power indicates 20% type II error (β). The large sample size was chosen, giving a sample size of 378. After adjusting for a 20% loss in follow-up, the final sample size becomes 472. Therefore, 236 pregnant women were needed in the intervention group and 236 pregnant women in the control group. A multi-stage sampling technique was used to select the study participants. In the first stage, two woredas (districts) were selected randomly from Ilu Aba Bor Zone. In the second stage, 16 kebeles were selected randomly from each selected district. The study participants were recruited from the selected kebeles. Initially, an enumeration of pregnant mothers in each selected kebele was performed. The number of pregnant women from the selected kebeles was determined using proportionate sampling techniques. A total of 472 pregnant women were selected using a simple random sampling technique and followed until the end of the study period (Fig. 1).

### Variables

Dependent variables are knowledge, attitude, and compliance level with iron-folic acid supplementation. Compliance with iron and folic acid supplementation is defined as the proportion of supplements taken as prescribed in relation to the number of days of supplementation, which was 70% or more of the prescribed supplement, which translates to taking the supplements at least 5 days per week based on a previous study [16].

### The intervention

After the baseline data collection, the participants were assigned to one of the two study arms: The intervention group or the comparison group. The intervention group received nutrition education, and the comparison group received the usual care during ANC. The nutrition education was given once a month for three sessions over three months. Each education session lasted for 45 to 60 min. The local language (Afan Oromo) was used for communication during the intervention delivery.

Training and educational tools were developed based on the Federal Ministry of Health training manual for trainers (Federal Democratic Republic of Ethiopia, 2013). Furthermore, the formative assessment at the beginning of the project guided the development of the tool. The intervention package included 1) a manual for the nutrition educators; 2) a training guide for the nutrition educators; and 3) Leaflets with the core messages for the pregnant women's families. The intervention protocol was pilot tested in a similar setting outside the study area for two weeks, and modifications were made based on the results of the pilot testing.

A health extension worker (HEWs) was recruited as educators. Educator was selected based on her prior expertise in providing educational services. A five-day intensive training was given to the educator using the training manual.

The intervention was implemented using two approaches. These include nutrition education for the intervention groups as well as monthly home visits and counseling for pregnant women to help them adopt the recommended practices after the nutrition education.

### Nutrition education for the intervention group

The intervention group was placed in groups and received nutrition education at nearby health posts once a month for three months. The strategy for delivering nutrition education sessions was guided by direct (teacher-directed) and interactive (discussion, sharing) teaching approaches. The main contents were as follows: (1) describing the importance of Iron and folic acid in promoting good health; (2) the consequences of inadequate intake of iron and folic acid, as well as vulnerability to the severity of the consequences of inadequate Iron and folic acid intake and (3) promoting adherence to the Iron and folic acid supplements.

### Intervention fidelity

The National Institutes of Health (NIH) Behavioral Change Consortium's best practice standards were used to evaluate the intervention's fidelity (Bellg et al., 2004). One of the proposals is: i) Research design: to build procedures for monitoring information contamination between treatment and comparison groups and reducing the likelihood of it happening, as well as for evaluating dose and intensity. In order to prevent information contamination, nonadjacent districts were selected. Treatment "dose" was adequately described and the same for each group in the trial, which also included a comparison group and a counseling manual; ii) Provider training: standardization of training to ensure that all trainers received the same training. As a result, instructors received instruction utilizing a manual, simulated counseling sessions, and ongoing supervision. iii) Treatment delivery: To make sure providers adhered to the treatment protocol, behavioral checks were made. In light of this, pre- and post-training assessments were used to evaluate educators' knowledge and abilities. iv) Receipt of treatment: implementation of the intervention was assessed using post-intervention knowledge, attitude, and compliance with IFA supplementation.

### Data collection tools and procedures

A pretested interviewer administered structured questions, including 11 on socio-demographic data, 9 on IFA supplementation knowledge, and 12 Likert scale questions on attitude, which were developed and used for data collection in this study. Baseline data on IFA knowledge, attitude, and compliance at the IFA level were collected. Then, after intervention, end-line data on IFA knowledge and attitude and compliance with IFA were collected. The level of knowledge about IFA supplementation during pregnancy was assessed using nine items: whether they have heard about IFA supplementation or not, benefits of IFA supplementation, frequency of use of IFAS, duration of taking IFA supplementation, side effects, management of side effects, effect of iron or folate deficiency, signs and symptoms of anemia, and food sources for iron during pregnancy. A correct answer for each item was scored as "1" and an incorrect answer was scored as "0". A summation of all the scores for each participant was done based on community-based nutrition education on iron and folic acid supplementation. Those who scored average value of 50% and above were considered as having good knowledge.

Attitude towards IFA supplementation was assessed on 12 Likert scale items. A correct answer for each item was recorded as 5, and an incorrect answer was scored as "1". The participants were considered to have a favorable attitude if they scored 70% or above and otherwise unfavorable [17]. Compliance with the IFA supplementation was assessed by pill count based on the number of remaining pills in the retained prescribed bottles or strips. The number of unused pills in the retained pill bottles or strips was counted and recorded at the last visit following the last week of the intervention. To ensure quality of data collection, training of four research assistants on research ethics and protocol and quality data collection was used.

### Data processing and analysis

Data were collected using Epicollect5, checked for clerical errors, and exported to SPSS version 22.0 for analysis. Descriptive statistics such as frequencies and percentages for discrete data and the mean values for continuous data were computed. Generalized estimating Equation (GEE) with a binary logit function was used to assess the effect of the intervention on knowledge, attitude, and compliance at the IFA level. First, we performed correlation on all structures, and because the Quasi-Information Criteria (QIC) for all correlation structures was the same, we used an exchangeable correlation structure. The model was run to account for clustered data and intra-subject observational correlation. The exchangeable working correlation structure was taken into account when fitting the model to account for the effects of various confounding variables. The study looked at socio-demographic and socioeconomic characteristics, household food security status, time, intervention, and time and intervention interaction. The intervention's effect was evaluated using time and intervention interaction. The odds ratio was calculated along with a 95% confidence level. A *p*-value of less than 0.05 was considered statistically significant.

## Results

### Socio-demographic characteristics of study participants

A total of 472 pregnant women participated in the study during the baseline, and 437 (92.6%) were in the study until the end. The majority (49.2%) of respondents were 21–25 years of age, with a mean age of 23.4 (SD = 3.7) years.98.1% of the study participants were married, and women who had a primary level of education were 51.5% (n = 243). Only 14% of study respondents were employed (*n* = 66). Whereas 7.8% (*n* = 37) of study participant husbands had attained tertiary level education, of that, 21.2% (*n* = 98) were employed. In terms of gravidity, most (*n* = 313, 66.3%) of the women were multigravida. The socio-demographic characteristics of respondents at baseline by study group are shown in (Table 1).

**Table 1:** Socio-demographic and obstetric characteristics of pregnant women who participated in the study in Ilu Aba Bor zone, Southwest Ethiopia, 2022

Variable	Category	Frequency	Percentage
Mother’s age categories	≤ 20 years	123	26.1
	21 to 25 years	232	49.2
	26 to 30 years	94	19.9
	31 to 35 years	23	4.9
Marital status	Married	463	98.1
	Others	9	1.9
Mothers’ educational status	No formal education	66	14.0
	Primary education	243	51.5
	Secondary education	136	28.8
	Tertiary education	27	5.7
Husband educational status	No formal education	43	9.1
	Primary education	203	43.0
	Secondary education	189	40.0
	Tertiary education	37	7.8
Mother’s Occupation	Gov. employee	7	1.5
	Merchant	12	2.5
	Daily laborer	36	7.6
	Student	12	2.5

### Effect of nutritional education on maternal knowledge of IFAS

**Fig. 2:**
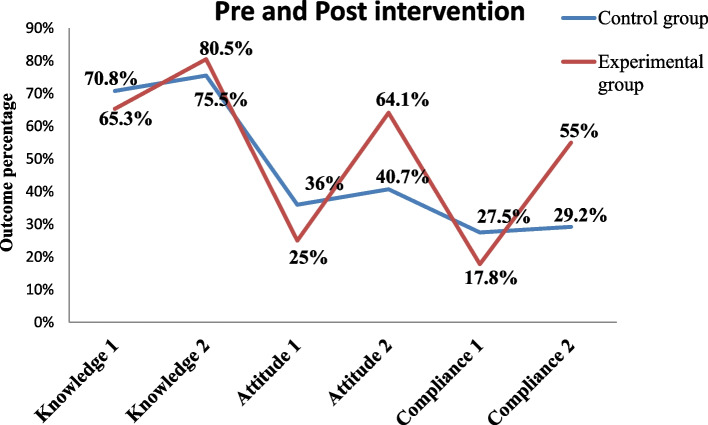
Pre and post nutritional education on Knowledge, Attitude and Compliance level of study participants. 1 pre intervention 2 post intervention

A comparison between baseline and end-line levels of maternal knowledge on IFA supplementation shows an increase of 15.2% in the intervention group (from 65.3% to 80.5%) as compared to 4.7% (from 70.8% to 75.5%) in the control groups (Fig. 2)*.* The intervention had a net effect of 10.5 percentage points (15.2–4.7) of improvement in IFA supplementation knowledge level. Therefore, the statistical significance of the difference in difference (DID) between the two groups was < 0.001. There was a significant change in levels of IFAS knowledge between the two time points, the odds of being somehow knowledgeable at end-line were 2.3 times that at baseline (AOR = 2.3: 95% CI 1.67–3.03), adjusting for other variables (Table 2).

**Table 2:** Factors associated with maternal knowledge on IFAS among pregnant women in rural communities of Ilu Aba Bor Zone, South West Ethiopia, 2022

Variables		*P*-value	AOR	[95% CI]	
				Lower	Upper
Nutritional education provided	Yes	< 0.001	2.3	1.67	3.03
	No		1		
Level of education	Tertiary Education	0.27	1.63	.688	3.864
	Secondary Education	0.61	1.16	.652	2.066
	Primary Education	0.19	1.41	.848	2.354
	No formal Education		1		
Current pregnancy	Unwanted	0.36	2.87	.293	28.077
	Missed Time	0.88	1.05	.582	1.873
	Planned		1		
Study Group	Experimental group	0.13	.73	.479	1.098
	Control group		1		
Interaction (group, time)	[Time = 2] *[Groups = 2]		1		
	[Time = 2] * [Groups = 1]	0.28	1.28	.812	2.027
Gravidity	Grand Multipara(V +)	0.55	1.53	.379	6.157
	Multipara(II-IV)	0.83	1.05	.669	1.652
	Primigravida		1		
Maternal age categories in years	31 – 35 years	0.02	9.54	1.417	64.255
	26 – 30 years	0.79	1.08	.609	1.909
	21 – 25 years	0.48	1.17	.759	1.796
	≤ 20 years		1		
	Housewife	0.21	4.85	.409	57.541
Mothers occupation	Student	0.41	3.11	.212	45.571
	Daily Laborer	0.08	9.29	.761	113.251
	Government employed		1		
	**Housewife**	**405**		**85.8**	
Husband Occupation	Employee	85		18.0	
	Merchant	33		7.0	
	Farmer	354		75.0	
Gravidity	Primigravida	143		30.3	
	Multipara (II–IV)	313		66.3	
	Grand multipara (V +)	16		3.4	
Pregnancy Status	Unwanted	8		1.7	
	Planned	427		90.5	
	Missed time	37		7.8	
ANC service visited	Yes	450		95.3	
	No	22		4.7	
Weeks of 1^st^ ANC Visit	≤ 4 weeks	62		15.3	
	5 – 8 weeks	47		9.9	
	9 – 16 weeks	267		56.6	
	17 – 27 weeks	83		17.6	
	≥ 28 weeks	3		0.6	
ANC service Provider visited	Nurse	21		4.4	
	Midwife	422		89.4	
	Health Officer	21		4.4	
	HEW	8		1.7	
Received nutritional counseling during ANC	Yes	191		40.5	
	No	281		59.5	
History of abortion	Yes	59		12.5	
	No	413		87.5	
Maternal Age (year)		**Mean**		**SD**	
		23.4		3.7	

### Effect of nutrition education on maternal attitudes towards IFA supplementation

The proportion of pregnant women who had a positive attitude towards IFA supplementation differs between the intervention and control groups, at 25% and 36%, respectively. The difference in attitude was statistically significant (*p* = 0.009). The proportion of pregnant women who had a positive attitude towards IFA supplementation among the experimental group after intervention was 64.1% and 40.7% in the control groups, which has a statistically significant difference across the study groups at a *P*-value of < 0.001.

There was a significant improvement in the proportion of pregnant women who had a positive attitude towards IFAS during the study period across the study groups. The increase was higher at 39.1 percentage points (from 25% to 64.1%) in the intervention group compared to 4.7 percentage points (from 36% to 40.7%) in the control group (Fig. 2). The intervention had a net effect of 34.4 percentage points (39.1–4.7) increase in positive attitude. Therefore, the statistical significance difference (DID) between the two groups was < 0.001 with a significant change in levels of positive attitude towards IFAS between the two time points; the odds of having a positive attitude at end-line were 5.6 times that at baseline (AOR = 5.6: 95% CI 4.01–7.85), adjusting for other variables (Table 3).

**Table 3:** Factors associated with maternal attitude on IFAS among pregnant women in rural communities of Ilu Aba Bor Zone, South West Ethiopia, 2022

Variables		AOR	[95% CI]	
			Lower	Upper
Nutritional education provided	Yes	5.6	4.01	7.85
	No	1		
Mother Knowledge on IFAS	Good knowledge	1.20	.85	1.71
	Poor knowledge	1		
Study Group	Experimental group	.61	.391	.944
	Control group	1		
Interaction (group, time)	[Time = 2] *[Groups = 2]	1		
	[Time = 2] * [Groups = 1]	1.27	1.09	1.48
Gravidity	Grand Multipara(V +)	1.91	.66	5.53
	Multipara (II-IV)	1.18	.742	1.88
	Primigravida	1		
Maternal age in years	31 – 35 years	1.04	.36	3.03
	26 – 30 years	1.33	.72	2.47
	21 – 25 years	1.04	.65	1.65
	≤ 20 years	1		
Mothers occupation	Housewife	1.37	.40	4.68
	Student	5.8	1.01	33.5
	Daily Laborer	5.99	1.65	21.82
	Government employed	1		

### Effect of nutrition education on maternal compliance with IFAS

In this study, respondents who took at least 70% (5 tablets) of the expected dose of IFAS tablets in the week preceding the interview were considered compliant with IFAS. There was an improvement in compliance with IFAS in both groups at the end line, which was a significant improvement in compliance with IFAS among the intervention group (from 18.2% to 55%). The intervention had a net effect of a 34.8% (36.5–1.7) increase in compliance. Moreover, it yielded a statistically significant difference since the DID between the two groups was statistically significant at a *p*-value < 0.001. There was significant improvement in the levels of IFAS knowledge at the end line. The odds of being in compliance with IFAS at end-line were 3.9 times higher than those at baseline (AOR = 3.9; 95% CI 2.67–5.57), adjusting for other variables (Table 4).

**Table 4:** Factors associated with maternal compliance with IFAS among pregnant women in rural communities of Ilu Aba Bor Zone, South West Ethiopia, 2022

Variables		AOR	[95% CI]	
			Lower	Upper
Intervention effect				
Intervention group	Yes	3.9	2.67	5.57
Comparison	No	1		
Level of education	Tertiary Education	1.07	.35	3.25
	Secondary Education	1.05	.52	2.12
	Primary Education	1.21	.63	2.33
	No formal Education	1		
Time of first ANC Visit	≤ 4 weeks	.49	.02	10.19
	5–8 Weeks	.82	.37	1.82
	9–16 Weeks	.38	.20	.73
	17–27 Weeks	.40	.15	1.11
	≥ 28 weeks	1		
Parity	Grand Multipara(V +)	1.57	.37	6.67
	Multipara (II-IV)	1.13	.62	2.05
	Primipara	1		
Maternal age in complete years	31 – 35 years	9.54	1.417	64.255
	26 – 30 years	1.08	.609	1.909
	21 – 25 years	1.17	.759	1.796
	≤ 20 years	1		
Mother Knowledge on IFAS	Good knowledge	1.75	1.17	2.62
	Poor knowledge	1		
Mother attitude towards IFAS	Positive Attitude	3.7	2.44	5.56
	Negative Attitude	1		

## Discussion

The aims of this study were to determine the effect of community-based nutritional education on knowledge, attitude, and compliance with IFA supplementation in the Ilu Aba Bor zone of southwest Ethiopia. This study identified that more than two-thirds of women have some knowledge regarding the benefits of IFA supplementation and the consequences of iron deficiency during pregnancy.

The findings indicate that community- based nutrition education resulted in a better improvement in knowledge in the intervention group as compared to the control group. The odds of being knowledgeable about IFA were 2.3 times higher among pregnant women who received nutrition education than those who did not. This shows that community nutritional education can contribute to a change in women's knowledge. Knowledge of pregnant women's attitudes towards IFA supplementation benefits and the consequences of not taking IFA supplementation can be improved through the delivery of well-organized and focused education at the community level by using community volunteers and health extension workers. Women's knowledge of the benefits of IFA supplementation is one of the factors that also affect their compliance with taking the supplement. This finding is in line with a study in Kiambu County, Kenya, which reported a higher improvement in women's knowledge of IFA among groups who were given health education on IFA than the control group [17]. Similarly, a study conducted in Indonesia and Pakistan reported that counseling offered at health facilities was found to be inadequate to increase maternal IFA knowledge as needed [18].

A pilot project in Vietnam, which employed community-based social mobilization and social marketing approaches in sites supported by volunteer village health workers and non-governmental organizations, also showed significant increases in the percentage of women with awareness that ‘poor nutrition led to anemia, that ‘weekly iron-folic acid supplementation could help to prevent anemia, and of the need for ‘more iron during pregnancy [19].

This study revealed a statistically significant improvement in attitude towards IFA supplementation in the intervention group when compared to the control group. These findings are similar to those of a nutrition education intervention study done among postpartum women that showed a significant improvement in overall positive health beliefs and the compliance of women to give up their negative beliefs, perceptions, and practices in relation to IFAS [20]. This result is similar to that of the study conducted in Kenya. In that study, although there was a higher improvement in attitude among the intervention group than the control group, the difference was not statistically significant. This change in attitude can be explained by the fact that as women learn about the benefits of IFA supplementation and the consequences of iron deficiency during pregnancy, there is a high possibility that they can develop a favorable attitude towards IFA supplementation.

Compliance with iron and folic acid was very low at baseline; almost one-fifth of pregnant women were taking more than five tablets a week. This finding is lower than studies conducted in Dire Dawa, Hawassa, and Addis Ababa [21–23]. This discrepancy could be due to the fact that those studies were conducted at health facilities, whereas our study was community-based.

The findings of this study show a significant improvement in the compliance level of IFA supplementation among women who received community-based nutritional education. In this study, the odds of compliance with IFA supplementation were 3.9 times higher among those women who received nutrition education than those who did not. This is in line with the study in Kenya, which resulted in improved compliance among women who received education by volunteers and community health workers compared to the control group, even though their results were not statistically significant.

Although we have tried to assess the effects of community-based nutrition education on women's knowledge, attitudes, and compliance with IFA using one of the best interventional study designs, this study is not without limitations. Due to the fact that in developing countries like Ethiopia, women seek antenatal care after the second half of pregnancy, we couldn’t find many women in their early gestation, which might have affected the result of the study. In addition, we only depend on the supplement provided by governmental health facilities, where sometimes drug stock outs have resulted in delays in receiving the supplement for small proportions of women in the study.

## Conclusion

The study revealed that community-based nutritional education can result in a significant change in knowledge, attitude, and compliance towards IFA supplementation, which supports the literature suggesting the importance of the intervention to overcome the problem of poor compliance and its associated consequences.

## Data Availability

The datasets generated and/or analysed during the current study are not publicly available due confidentiality but are available from the corresponding author on reasonable request.
